# BLIMP-1 Mediated Downregulation of TAK1 and p53 Molecules Is Crucial in the Pathogenesis of *Kala-Azar*

**DOI:** 10.3389/fcimb.2020.594431

**Published:** 2020-10-29

**Authors:** Gundappa Saha, Adarsh Kumar Chiranjivi, Bakulesh M. Khamar, Kumari Prerna, Manish Kumar, Vikash Kumar Dubey

**Affiliations:** ^1^ Department of Biosciences and Bioengineering, Indian Institute of Technology Guwahati, Guwahati, India; ^2^ Research & Development, Cadila Pharmaceuticals Limited, Ahmedabad, India; ^3^ School of Biochemical Engineering, Indian Institute of Technology Banaras Hindu University (BHU), Varanasi, India

**Keywords:** Leishmaniasis, *kala-azar*, immuno-suppression, BLIMP-1, macrophage

## Abstract

Precise regulation of inflammasome is critical during any pathogenic encounter. The whole innate immune system comprising of pattern recognition receptors (PRRs) relies on its ability to sense microbes. The fate of cellular death in infected cells depends mostly on the activation of these inflammasome, the dysregulation of which, due to functional manipulation by various pathogens, leads to be the cause of many human diseases. Here, an interesting finding has been observed which is related to how *Leishmania donovani* parasites exploit various host mediator molecules to cause immunosuppression. Here we report for the first time that the parasites check pyroptosis in the infected cells *in-vitro* by BLIMP-1 mediated suppression of TAK1 and p53 proteins. This might be one of the reasons how parasites evade the pro-inflammatory response of the host cells. Further understandings and validations are required to come up with better therapeutic approaches against *kala-azar*.

## Introduction

Macrophages are the sentinel immune cells that come in response to pathogenic encounter and direct the innate immune response toward adaptive immune system. The innate immune system relies on the pattern recognition receptors (PRRs) to sense any microbial infection or tissue damage and finally initiate inflammatory reactions by activation of various inflammasomes depending upon the types of pathogen (Newton and Dixit, 2012). Prior to its activation, priming leads to the activation of NFκB and subsequent expression of inflammasome components and the pro-forms of the inflammatory cytokines, IL-1β and IL-18. Apart from this, priming also modulate the post-translational modifications (PTMs) of NLR family pyrin domain containing 3 (NLRP3), apoptosis-associated speck like protein containing a caspase recruitment domain (ASC) and pro-caspase 1 to facilitate the assembly of the inflammasome and thus activation (Yang et al., 2016). Among these modifications, phosphorylation and ubiquitination are the most characterized ones in the regulation of inflammasome signaling, catalyzed by hundreds of enzymes (Khoury et al., 2011). The highly diverse and versatile PTMs play an important role in shaping the dynamics of inflammatory response during pathogenic infections and thus remain an exciting area of research for the foreseeable future. As per previous report in terms of fungal infections, NLRP3 and ASC, both have been reported to be phosphorylated by Syk kinase and many other kinases (Gross et al., 2009). Recently, a threonine-serine kinase, NIMA Related Kinase 7 (NEK7) was identified by many independent groups to be involved in the activation of NLRP3 inflammasome signaling (He et al., 2016). NEK7 has been reported as NLRP3 binding protein which helps in the regulation of NLRP3 oligomerization and activation depending on potassium efflux. Also, intracellular reactive oxygen species (ROS) formation from the mitochondria helps NEK7 to efficiently bind with NLRP3. Whereas, through screening of natural products, TGF-β activated kinase-1 (TAK1) was also identified as a kinase regulating the NLRP3 inflammasome by phosphorylation (Gong et al., 2010). In contrast, recent report says, TAK1 controls NLRP3 inflammasome homeostasis by restraining inflammation and cell death in myeloid cells (Malireddi et al., 2018). So, the role of TAK1 in the regulation of inflammasome is still elusive and needs further investigations.

Apart from enzymes involved in the regulation of PTMs of NLRP3 inflammasome, the role of p53—a tumor suppressor gene in inflammasome regulation cannot be ruled out. Sanjeev et al., showed that caspase 1 is transcriptionally activated by p53 suggesting its role in inflammation (Gupta et al., 2001). The involvement of p53 gene in various non-canonical cell death pathways has also been discussed apart from apoptosis (Ranjan and Iwakuma, 2016). There is a similar mechanistic pathway that exists for both p53 and NFκB activation. The murine double-minute 2 (Mdm2) or human murine double-minute 2 (hMdm2) is an E3 ubiquitin ligase that binds to p53 molecule in normal cells and upon stress stimuli, the degradation of Mdm2 allows p53 stabilization, activation, and its cellular response (Moll and Petrenko, 2003). On the other hand, NFκB subunits are held by inhibitor of nuclear-κB (IκB) in the cytoplasm and upon stimulation, IκB gets phosphorylated by TAK-1 mediated inhibitor of nuclear factor-κB kinase (IKK) leading to its proteasomal degradation (Adhikari et al., 2007). This allows NFκB to translocate into the nucleus for transcription of various pro-inflammatory genes involved in inflammation (Liu et al., 2017). According to a previous report, p53 and NFκB cross-talk antagonistically in signaling pathway even though they have similar activation mechanism (Huang et al., 2007). But there are reports available wherein it has been shown that both of them co-regulate the pro-inflammatory axis of cell death pathways in macrophages (Ryan et al., 2000; Lowe et al., 2014). So, the interaction between p53 and NFκB is different depending upon the type of cells and cellular microenvironment.

Here, we have reported for the very first time the cross-talk between NEK7, TAK1, NFκB, and p53 in the context of B-cell induced maturation protein 1 (BLIMP-1) overexpression during *Leishmania donovani* infection (Montes De Oca et al., 2016). BLIMP-1 is a zinc finger containing DNA binding transcriptional repressor of IFN-β (Huang, 1994) and serves as a master regulator of antibody-producing B-cells, orchestrating T-cell homeostasis (Fu et al., 2017). The role of BLIMP-1 in regulating macrophage pyroptosis during *L. donovani* infection has already been justified previously (Saha et al., 2019a) and in this study how NEK7, TAK1, NFκB, and p53 are cross-regulating the pyroptotic cell death pathway have been explored during infection. Our previous report on the impairment of caspase 1 dependent NLRP3 inflammasome activation during *L. donovani* infection (Saha et al., 2019b) has been further justified by finding out the roles of PTM enzymes in activation of the inflammasome. The data supports the fact that parasites exploit BLIMP-1 upregulation to suppress the expression of TAK1 and p53, thereby tweaking the tightly controlled NFκB-NLRP3 signaling pathway, inhibiting the intracellular ROS formation and evasion of pyroptosis during infection.

## Materials and Methods

### Cell Culture and Other Materials

Briefly, THP-1 and J774A.1 were procured from National Centre for Cell Sciences, Pune and cultured at 37°C and 5% CO_2_ in Roswell Park Memorial Institute (RPMI-1640) media and Dulbecco’s Modified Eagle’s Medium (DMEM) respectively supplemented with 10% FBS, 2 mM glutamine, 100 U/ml penicillin, 100 µg/ml streptomycin, and 2.5 μg/ml amphotericin B. Phorbol 12-myristate 13-acetate (PMA) was used at a concentration of 100 ng/ml to differentiate monocytic THP-1 cells into macrophages at 37°C and 5% CO_2_ overnight. *L. donovani* promastigotes (*Ld*/BHU1081 strain) were received as a kind gift from Shyam Sundar, Banaras Hindu University, India, and cultured in BSL-II and maintained at 28°C in M199 liquid media supplemented with 10% fetal bovine serum (FBS), 100 U/ml penicillin, and 100 µg/ml streptomycin. Antibodies like NEK7 and TAK1 were procured from Santa Cruz while other antibodies like α-tubulin, p53 and Cyt c were procured from Bio-Bharti and Thermo, respectively.

### Real-Time PCR

Total RNA was isolated using RNeasy Mini Kit (QIAGEN) protocol and proceeded with cDNA preparation using ProtoScript^®^ First Strand cDNA Synthesis Kit (New England Biolabs) after DNase treatment for 1 h at 37°C. The mRNA levels of *nek7* and *tak1* were analyzed using specific primers in qRT-PCR. For all the qRT-PCR experiments, *β-actin* gene was used as endogenous housekeeping control to normalize the relative fluorescence signal of the target genes. All the primers sequences used are listed in **Table 1**.

**Table 1:** List of primer sequences used for real-time PCR experiments.

S. No.	Primer Name	Sequence (5’–3’)
1	*nek7*_FP	TGGATGAGCAATCACAAGGAAT
2	*nek7*_RP	CCGGTCGTAAGGCCTTCTG
3	*tak1*_FP	GCGTCGGAAACCCTTTGA
4	*tak1*_RP	TGAACAGCCCACATGATTCG
5	*β-actin*_FP	CTGGCACCCAGCACAATG
6	*β-actin*_RP	GCCGATCCACACGGAGTACT

### siRNA-Mediated Knock Down

BLIMP-1 knock down was carried out using *blimp-1* specific siRNA procured alongwith a *scrambled* siRNA from Invitrogen. Briefly ~2 × 10^5^ cells (THP-1 and J774A.1) were seeded in RPMI-1640 media and DMEM, respectively in 35-mm petri dishes and incubated at 37°C, 5% CO_2_ for 24 h. PMA (100 ng/ml) was used for THP-1 cells to differentiate into macrophages. Serum-free Opti-MEM media was used to prepare siRNA-lipid mix and incubated the cells for 6 h followed by addition of complete media and finally incubated at 37°C, 5% CO_2_ for another 18 h. The effect of siRNAs (30 nM) used for knock-down was validated using western blot analysis (data not shown).

### Promastigote Infection

Live *L. donovani* promastigotes were used to infect the differentiated macrophage cell lines at MOI of 1:5. The cells were counted in haemocytometer and resuspended in respective media (RPMI-1640 for THP-1 and DMEM for J774A.1 cells) and incubated for 24 h at 37°C, 5% CO_2_. For all siRNA knock down experiments, infection was done after siRNA treatment.

### Immunoblotting

Briefly, 2 × 10^5^ cells/experimental sample of THP-1 and J774A.1 were used to lyse the cells using RIPA buffer containing 1× Protease Inhibitor Cocktail, 1 mM PMSF, and 10 mM EDTA. An equal amount (25 μg/lane) of cell lysates of different samples were run on a 12% SDS-PAGE followed by transfer into a polyvinylidene fluoride (PVDF) membrane. Blocking was done using 5% skimmed milk for 2 h at room temperature (RT) followed by washing with 1× TBST buffer thrice. Then, primary antibodies for NEK7 (1:100 dilution), TAK1 (1:100 dilution), p53 (1:1,000 dilution) and Cyt c (1:750 dilution) were used for incubating the membrane at 4°C overnight. Next, the membrane was washed again with 1× TBST and incubated with secondary IgG antibody conjugated with HRP at for 45 min at room temperature. The membrane was washed with 1× TBST and chemiluminescent HRP substrate was used for band detection under a Chemi-Doc.

### Immunofluorescence Using Flow Cytometry

Cells were trypsinized using 1× Trypsin, centrifuged and washed with cold 1× PBS twice prior to flow cytometry analyses. Cells were fixed with 4% paraformaldehyde for 5 min at RT and then permealized using 0.01% Triton-X 100 for 5 min at RT. Blocking was done with 3% bovine serum albumin (BSA) for 2 h at RT and then was incubated with primary antibodies (TAK1 and p53) at dilution of 1:50 at 4°C overnight. Then, cells were incubated with secondary antibody conjugated with FITC was used (1:500 dilution) for 1 h at RT. Cold 1× PBS were used in all the intermediate and final steps to wash the cells thrice. Cells were resuspended finally in 1× PBS to process them in flow cytometer. Histogram plots shown in **Supplementary Figures 1 and 2**.

### Statistics and Densitometry Analyses

The western blot data were analyzed using densitometry using Image J software where a normalization factor was calculated for the loading control (α-tubulin) to normalize the signals of the target proteins. A statistical data analysis was determined by unpaired t-test in Sigma Plot 10 software using different biological replicates for all the experiments and has been represented by mean ± SEM. A “p” value of less than 0.05 has been considered significant. All replicate blots are shown in **Supplementary Figures 3–5**.

## Results

### Downregulation of NEK7 and TAK1 During Infection

**Figure 1:**
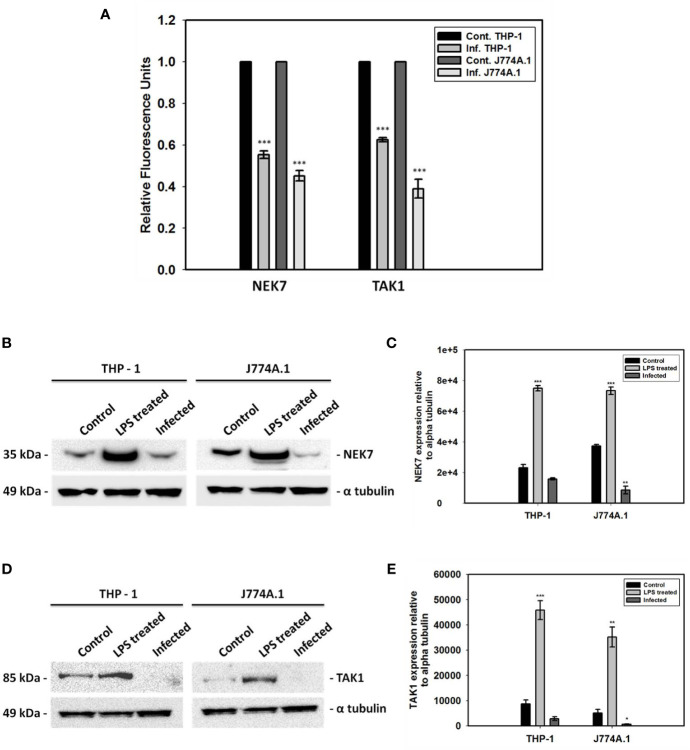
Gene expression analyses and immunoblotting of NEK7 and TAK1. **(A)** Transcriptional analyses of two kinases, *nek7* and *tak1* involved in the PTMs of NLRP3 inflammasome have been shown in both THP-1 and J774A.1 cells infected with promastigotes at MOI of 1:5 for 24 h; Decrease in **(B, C)** NEK7 expression and **(D, E)** TAK1 expression was found in both THP-1 and J774A.1 cells infected with promastigotes at MOI of 1:5 for 24 h. Bacterial LPS treated cells were used as positive controls for the experiment. (Note: n = 3, where n represents three different independent experimental replicates for all the experiments; * denotes p < 0.05, ** denotes p < 0.005, *** denotes p < 0.001).

NEK7 is very crucial in phosphorylating the NLRP3 component and thereby activating the inflammasome in the presence of higher intracellular ROS level. On the other hand, TAK1 is a significant kinase playing a role in activating NLRP3 inflammasome *via* NFκB activation. However, significant decrease in the mRNA level of *nek7* and *tak1* during *L. donovani* infection in both THP-1 and J774A.1 cell lines have been found (**Figure 1A**). The transcriptional downregulation of both *nek7* and *tak1* have been correlated with their decrease in the protein expression level in the infected cells as compared to control uninfected and LPS treated cells (**Figures 1B–E**).

### Resumption of TAK1 Expression in Blimp-1 Deficient Infected Cells

**Figure 2:**
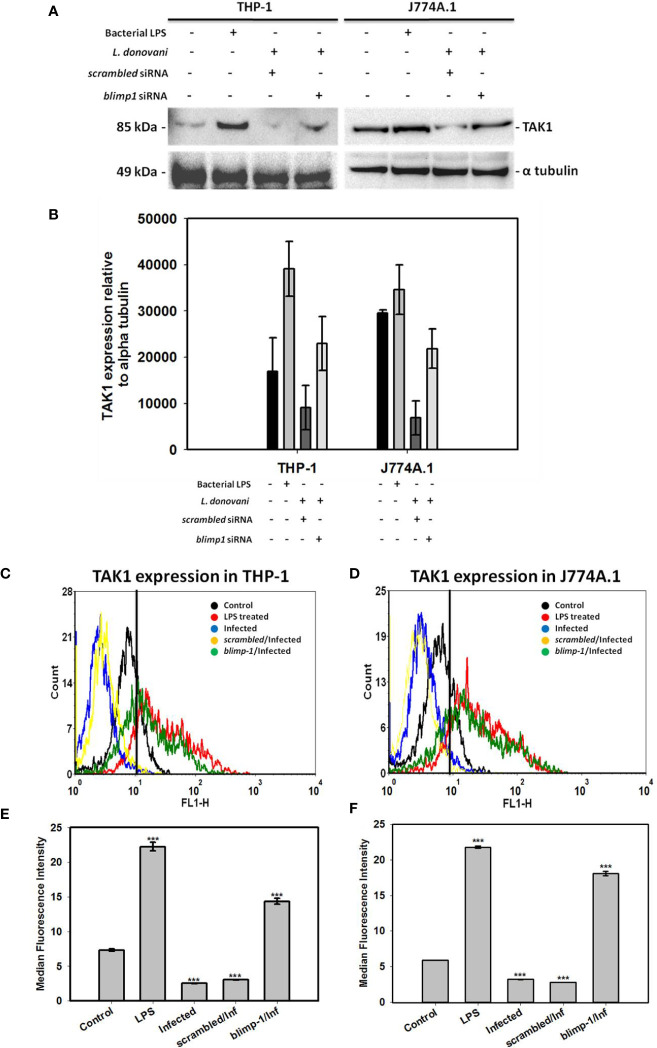
Quantification of TAK1 expression in *blimp-1* deficient cells infected with promastigotes at MOI of 1:5 for 24 h in both THP-1 and J774A.1 cell lines were measured using immunoblotting and flow cytometry. Bacterial LPS treated cells were used as positive controls for the experiment; **(A)** Western Blot, **(B)** Densitometry analyses of TAK1 expression. For flow cytometry, overlaid histogram plot was shown for **(C)** THP-1 cells and **(D)** J774A.1 stained cells. Median Fluorescence Intensity (MFI) has also been plotted for **(E)** THP-1 and **(F)** J774A.1 cells. (Note: n = 2 for **(A, B)** and n = 3 for **(C–F)**, where n represents different independent experimental replicates for all the experiments; *** denotes p < 0.001; percentage of gated cells is 100% and total event counted is 2500 for all flow cytometry experiments).

We thought of assessing the expression of both NEK7 and TAK1 in *blimp-1* deficient cells infected with promastigotes since BLIMP-1 has been found to be a key player in the pathogenesis of *kala-azar* (Saha et al., 2019a). Interestingly, we found BLIMP-1 over-expression to be involved with only TAK1 regulation as a repressor because TAK1 expression has been resumed in *blimp-1* deficient cells infected with promastigotes (**Figures 2A, B**). The same has been further verified by flow cytometry experiment where cells were stained more with TAK1 antibody in *blimp-1* deficient cells infected with promastigotes as compared to infection control (**Figures 2C, D**). This resumed staining of cells is comparable with LPS treated cells and has been shown by median fluorescence intensity (MFI) (**Figures 2E, F**). On the other hand, no such resumption or changes were found in NEK7 expression pattern in *blimp-1* deficient cells infected with the parasite and lacked consistency. Thus, we can attribute our finding related to BLIMP-1 induced TAK-1 repression also might be due to some indirect effect through other signaling pathways. These findings advocate us to dig deeper into the mechanisms of how BLIMP-1 is regulating the TAK1 mediated pathway of NFκB activation.

### BLIMP-1 Negatively Regulates p53 Expression

**Figure 3:**
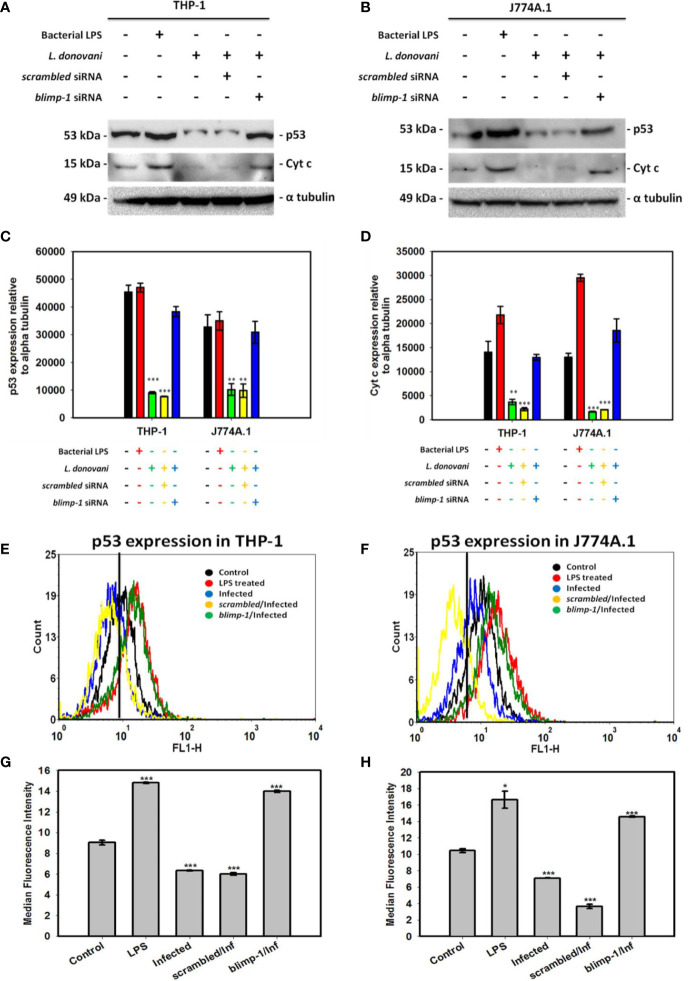
Quantification of p53 expression in *blimp-1* deficient cells infected with promastigotes at MOI of 1:5 for 24 h in both THP-1 and J774A.1 cell lines were measured using immunoblotting and flow cytometry. Immunoblot of p53 and Cyt c were shown in **(A)** THP-1 cells and **(B)** J774A.1 cells along with densitometry analyses in **(C)** THP-1 and **(D)** J774A.1 cells. For flow cytometry, overlaid histogram plot for p53 was shown in **(E)** THP-1 cells and **(F)** J774A.1 stained cells. Median Fluorescence Intensity (MFI) has also been plotted in **(G)** THP-1 and **(H)** J774A.1 cells.(Note: n = 3, where n represents three different independent experimental replicates for all the experiments; * denotes p < 0.05, ** denotes p < 0.005, and *** denotes p < 0.001; percentage of gated cells is 100% and total event counted is 2,500 for all flow cytometry experiments).

Despite our vast knowledge about multifunctional role of p53, its involvement in the host defense against pathogenic infections remains unknown. Interestingly, we found reduced expression of p53 level in the *Leishmania* infected cells and later resumed in *blimp-1* deficient cells infected with promastigotes as compared to controls by immunoblot (**Figures 3A–D**). In agreement with this, flow cytometry analyses also demonstrate that cells were stained more with p53 antibody in *blimp-1* deficient cells infected with promastigotes as compared to infection control (**Figures 3E–H**). This data can be attributed with a recent finding about how BLIMP-1 regulates the control of cell proliferation and survival through the negative regulation of p53 at the transcriptional level (Yan et al., 2007).

### BLIMP-1 Dependent Reduced Cytochrome C Expression During Infection

The involvement of p53 expression in mediating non-canonical forms of cell death has been explored and mentioned earlier (Gupta et al., 2001). The reduced intracellular ROS level during infection as per our previous results (Saha et al., 2019b) can also be explained in a way that p53 induces the level of BAX protein (pro-apoptotic) followed by increase in cytochrome c (Cyt c) level and thereby formation of intracellular ROS by the mitochondria which helps in either pathogen clearance or death of infected cells (Ranjan and Iwakuma, 2016). In contrast, there is reduced level of p53 in the infected cells leading to decreased Cyt c formation in the infected cells (**Figures 3A–D**). Moreover, the level of p53 and Cyt c has been shown to be resumed in *blimp-1* deficient cells infected with promastigotes suggesting an antagonistic role of BLIMP-1 on p53 dependent activation of cell death (Yan et al., 2007).

## Discussion

Recent reports suggest that pathogens, especially those with an intracellular life cycle, modulate the human host cell to ensure their own survival (Eisenreich et al., 2019; Thakur et al., 2019). However, they also need to counteract the severe damage that infections often cause to the host in order to avoid the immediate loss of their replicative niche. The cumulative role of NEK7 and TAK1 in a controlled manner has been reported earlier in initiating the activation of NLRP3 inflammasome and thereby causing caspase 1 mediated pyroptosis (He et al., 2016; Malireddi et al., 2019). In addition, DNA damage and ROS being strong inducers of p53-mediated apoptosis, bacterial pathogens along with few virus and protozoan parasites have adopted various mechanisms to escape and bypass p53 signaling to ensure host cell survival, highlighting the importance of this pathway for intracellular pathogens (Zaika et al., 2015; Miciak and Bunz, 2016). Here, we report for the first-time reduced level of NEK7, TAK1 and p53 expression during *L. donovani* infection leading to decrease in Cyt c release wherein the expression of these mediators (TAK1, p53 and Cyt c) except NEK7were resumed significantly and consistently in *blimp-1* deficient cells infected with promastigotes.

Transcriptional and translational downregulation of NEK7 and TAK1 level in infected cells as compared to control was an interesting finding which led us to design further experimentations to validate the role of NEK7 and TAK1 regulation in host immune surveillance during infection. In order to assess their roles, we thought of analyzing the expression level of NEK7 and TAK1 in *blimp-1* deficient cells infected with promastigotes since we found BLIMP-1 as a potential host molecule aiding parasite to evade the host immune response in our previous report (Saha et al., 2019a). To our surprise, TAK1 and not NEK7 expression level was found to be resumed significantly and consistently in *blimp-1* deficient cells infected with promastigotes as compared to controls. This data was conclusive enough to support the fact that these parasites are exploiting a major host molecule (BLIMP-1) in order to suppress the pro-inflammatory environment inside the cells and thereby inhibiting/downregulating a molecule (TAK1) upstream of NFκB activation pathway. In addition, we found reduced level of p53 and Cyt c in infected cells and their resumptions in *blimp-1* deficient cells infected with promastigotes as compared to controls were another findings which can be attributed and linked with our previous reports (Saha et al., 2019a; Saha et al., 2019b). In another report, this fact has already been established previously that BLIMP-1 represses p53 transcription by binding to its promoter and thereby provides a mechanistic explanation of induced p53 expression level in BLIMP-1 depleted cells (Yan et al., 2007). So, the decrease in p53 and Cyt c level might be one of the reason behind interruption ofcanonical as well non-canonical cell death pathways in infected cells. Thus, we can hypothesize that the parasites actually inhibit pyroptosis (non-canonical cell death pathway) by downregulation of p53 which is a transcriptional activator of human caspase 1 (Gupta et al., 2001). The reason behind the negative regulation of these mediators by BLIMP-1 might be an indirect effect of some other signaling pathways during infection.

Summarizing the cross-talks among all the host mediators in NLRP3 inflammasome driven cell death pathway, we can conclude that TAK1 induces the activation of NFκB followed by NLRP3 inflammasome activation. This triggering of cellular environment results in p53 stabilization which induces Cyt c release resulting in increased production of intracellular ROS favoring NLRP3 inflammasome activation by facilitating NLRP3 phosphorylation by NEK7. The downstream effect of NLRP3 inflammasome activation causes cells to undergo caspase 1 dependent pyroptosis, releasing IL-1β and IL-18 (Saha et al., 2019b). The above cross-regulation of pathways gets halted during *L. donovani* infection *in-vitro* by over-expression of BLIMP-1 protein. This BLIMP-1 was already found to directly repress p53 (Yan et al., 2007) whereas there might be some indirect mechanism of how TAK1 is being repressed by BLIMP-1 *via* other signaling pathways. In a nutshell, we conjecture that the parasites control pyroptosis in the infected cells by BLIMP-1 mediated suppression of TAK1 and p53 which impairs the NFκB – NLRP3 inflammasome signaling affecting the pro-inflammatory response due to decreased IL-1β processing as discussed in our early report (Saha et al., 2019a; Saha et al., 2019b). This work is a significant contribution to understand the poorly understood mechanism of *Leishmania* induced BLIMP-1 exploitation for evading the inflammatory regulation of the host cells. Although, detailed and more experimentations on *in-vivo* animal models will further enrich our knowledge on this topic. Thus, interesting facts have been observed on how *L. donovani* parasites modulate various host cell signaling pathways and exploit mediator molecules to cross-talk between different pathways for their survival and progression of infection.

## Data Availability Statement

The raw data supporting the conclusions of this article will be made available by the authors, without undue reservation.

## Author Contributions

GS has done experiments, acquisition, analysis, interpretation of data, and wrote the paper. AC has done flow cytometry experiments and helped in data analysis of the same. KP did scientific editing of the manuscript. VKD, MK, and BMK have helped in conceptualizing the idea and interpretation of data. All authors contributed to the article and approved the submitted version.

## Conflict of Interest

BMK was employed by the company Cadila Pharmaceuticals Limited, India.

The remaining authors declare that the research was conducted in the absence of any commercial or financial relationships that could be construed as a potential conflict of interest.
