# Supplementary material for: BLIMP-1 Mediated Downregulation of TAK1 and p53 Molecules Is Crucial in the Pathogenesis of Kala-Azar
Source: Front Cell Infect Microbiol. 2020 Oct 29;10:594431. doi: 10.3389/fcimb.2020.594431 (PMC7658262; doi:10.3389/fcimb.2020.594431)
Supplement: Supplementary file 1 [file DataSheet_1.pdf]

BLIMP-1 mediated downregulation of TAK1 and p53 molecules is crucial in the pathogenesis of *kala-azar*

Gundappa Saha[1], Adarsh Kumar Chiranjivi[1], Bakulesh M. Khamar[2], Kumari Prerna[3], Manish Kumar[1] and Vikash Kumar Dubey[3*]

[1]*Department of Biosciences & Bioengineering, Indian Institute of Technology Guwahati, Assam - 781039, India,*

[2]*Cadila Pharmaceuticals Limited, Ahmedabad, Gujarat - 382210, India,*

[3]*School of Biochemical Engineering, Indian Institute of Technology BHU, Varanasi, UP – 221005, India*

* Corresponding author: *Prof. Vikash Kumar Dubey, School of Biochemical Engineering, Indian Institute of Technology BHU, Varanasi; Email ID: vkdubey.bce@iitbhu.ac.in*

Supplementary Figure 1

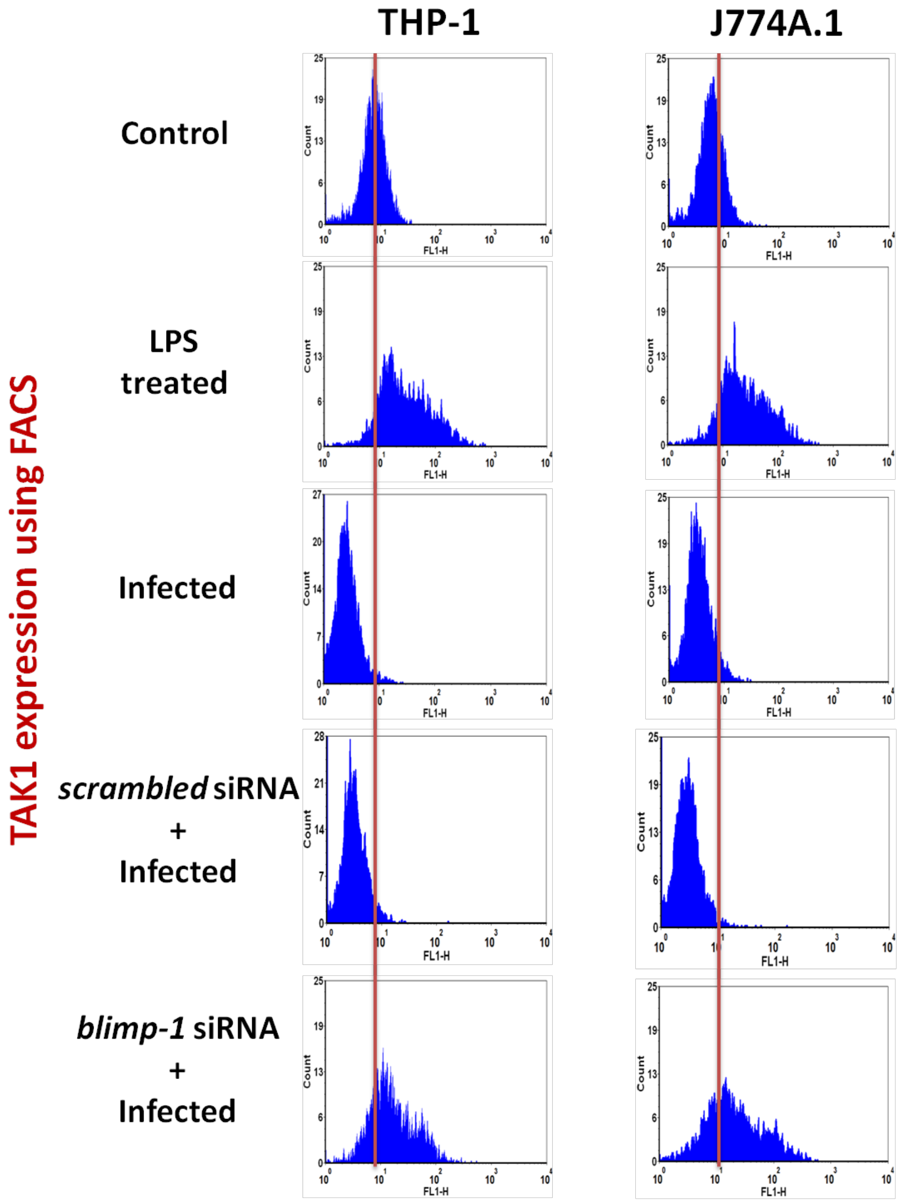

Supp. Fig. 1: Histogram plot for individual samples representing TAK1 expression in THP-1 and J774A.1 cells. BD FACS Calibur was used to run the samples and green fluorescence of FITC was measured in FL1-H channel. FCS Express 5 software was used to analyze the data using histogram plots and finally overlaid histogram plot was shown in the main manuscript. Median Fluorescence Intensity (MFI) has also been plotted for the samples to statistically justify the mean fluorescence emitted by the stained cells.

Supplementary Figure 2

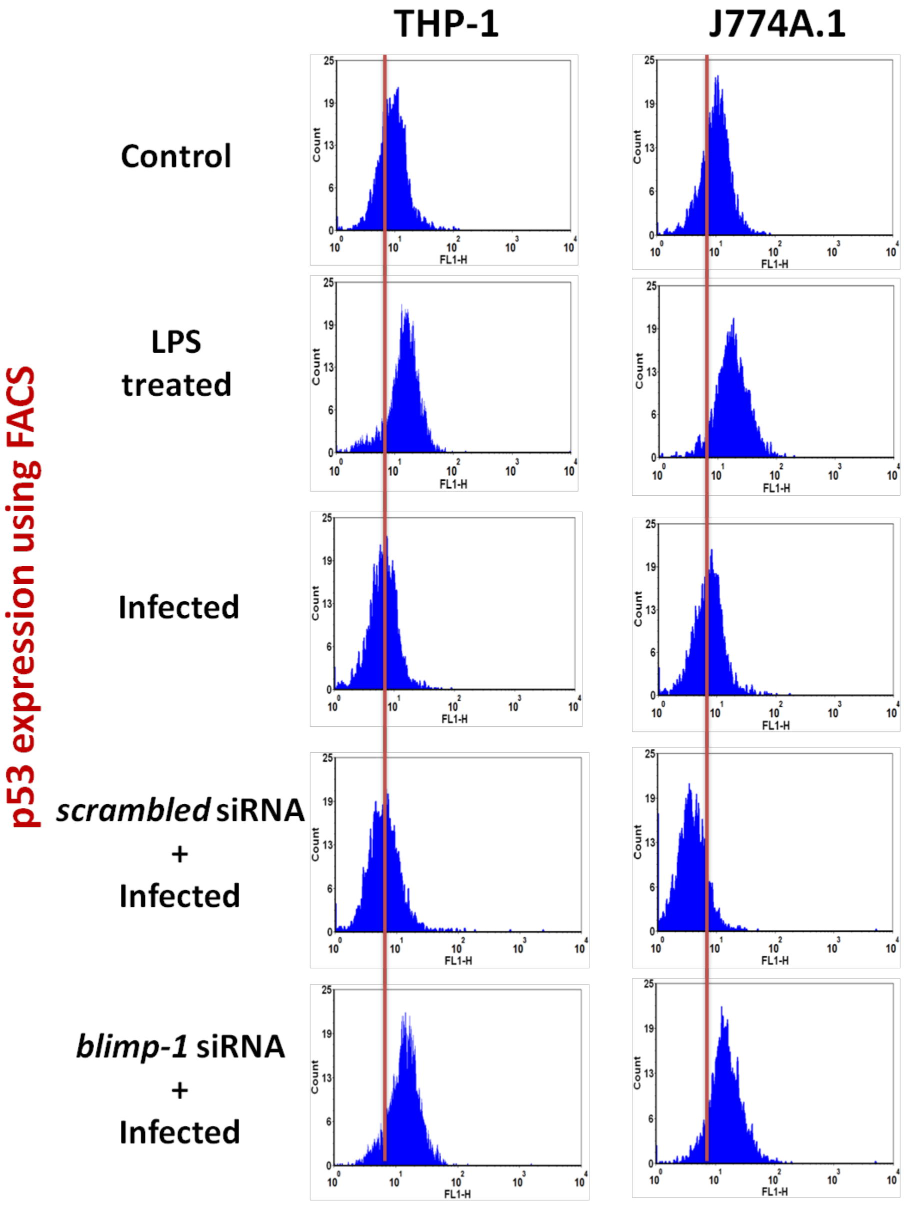

Supp. Fig. 2: Histogram plot for individual samples representing p53 expression in THP-1 and J774A.1 cells. BD FACS Calibur was used to run the samples and green fluorescence of FITC was measured in FL1-H channel. FCS Express 5 software was used to analyze the data using histogram plots and finally overlaid histogram plot was shown in the main manuscript. Median Fluorescence Intensity (MFI) has also been plotted for the samples to statistically justify the mean fluorescence emitted by the stained cells.

Supplementary Figure 3

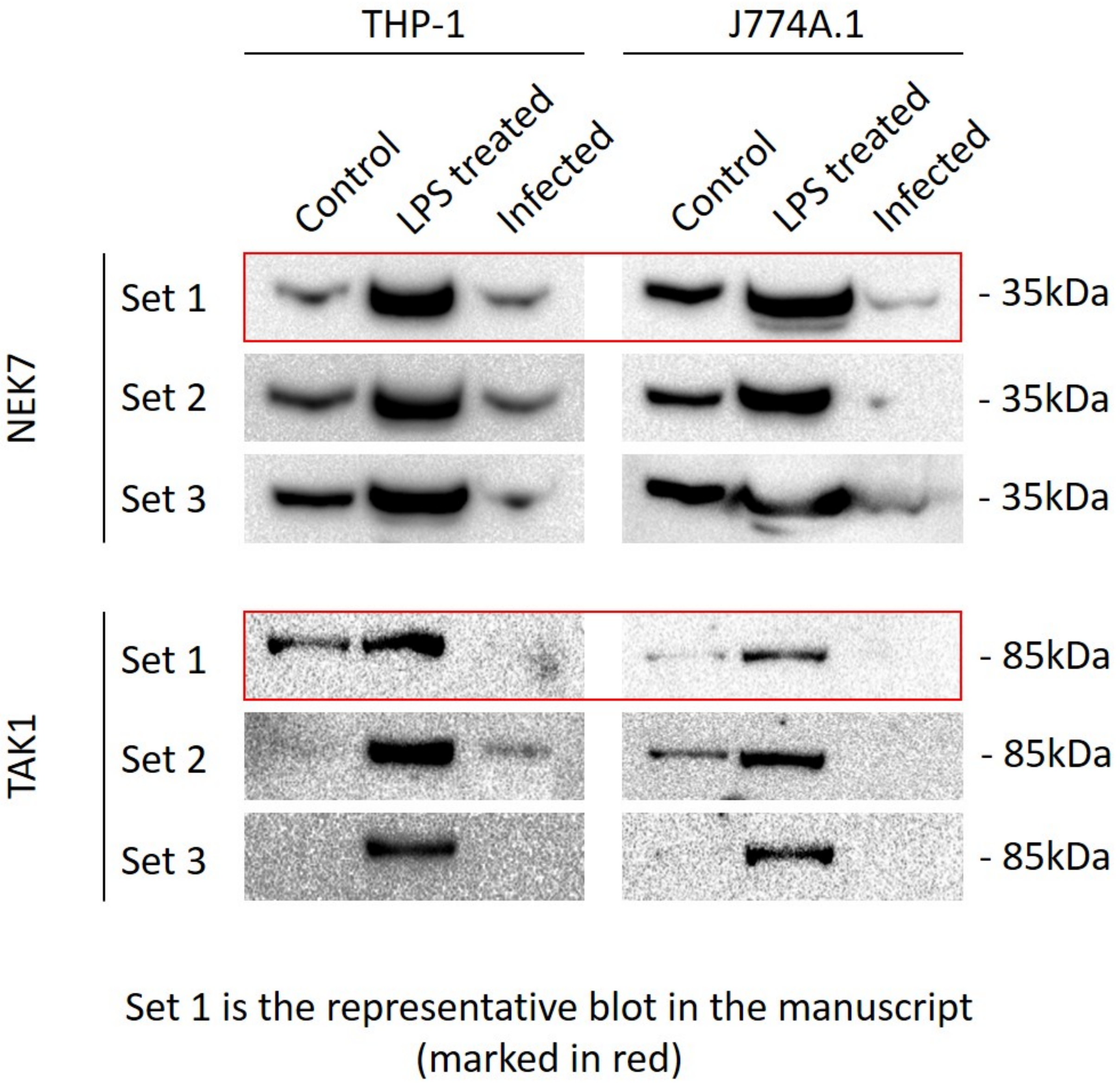

Supp. Fig. 3: Different replicate blots were shown for manuscript figure 1. Red marked (Set 1) was used in the manuscript as representative blot. (Note: n=3)

Supplementary Figure 4

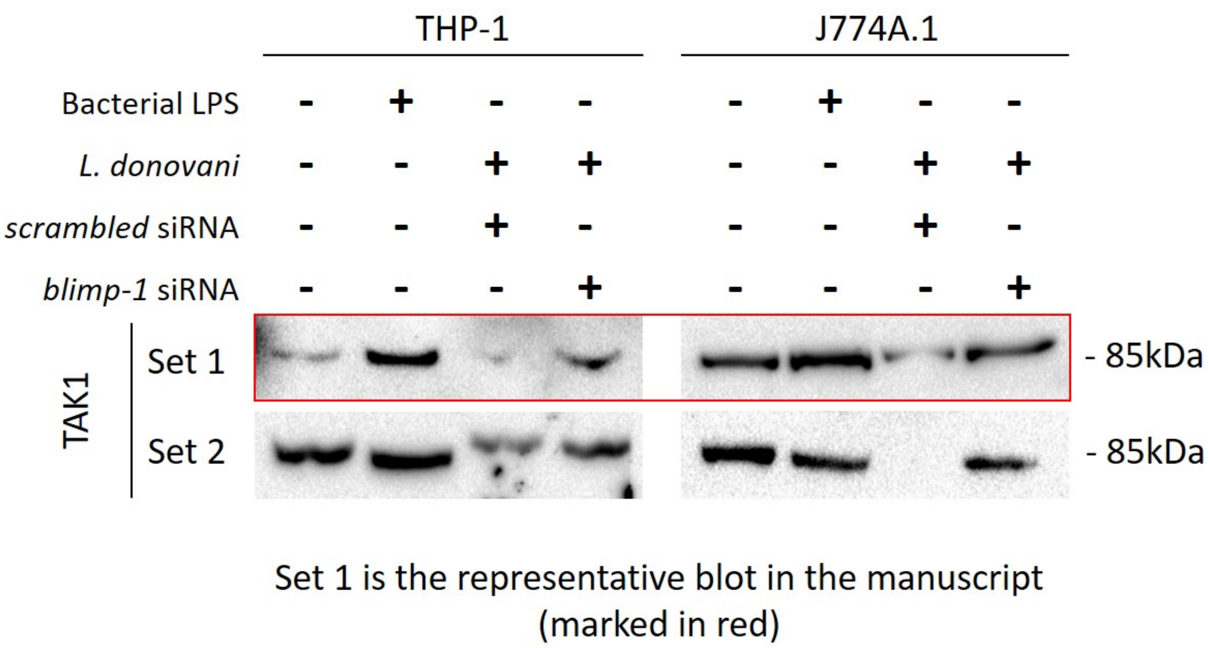

Supp. Fig. 4: Different replicate blots were shown for manuscript figure 2. Red marked (Set 1) was used in the manuscript as representative blot. (Note: n=2)

Supplementary Figure 5

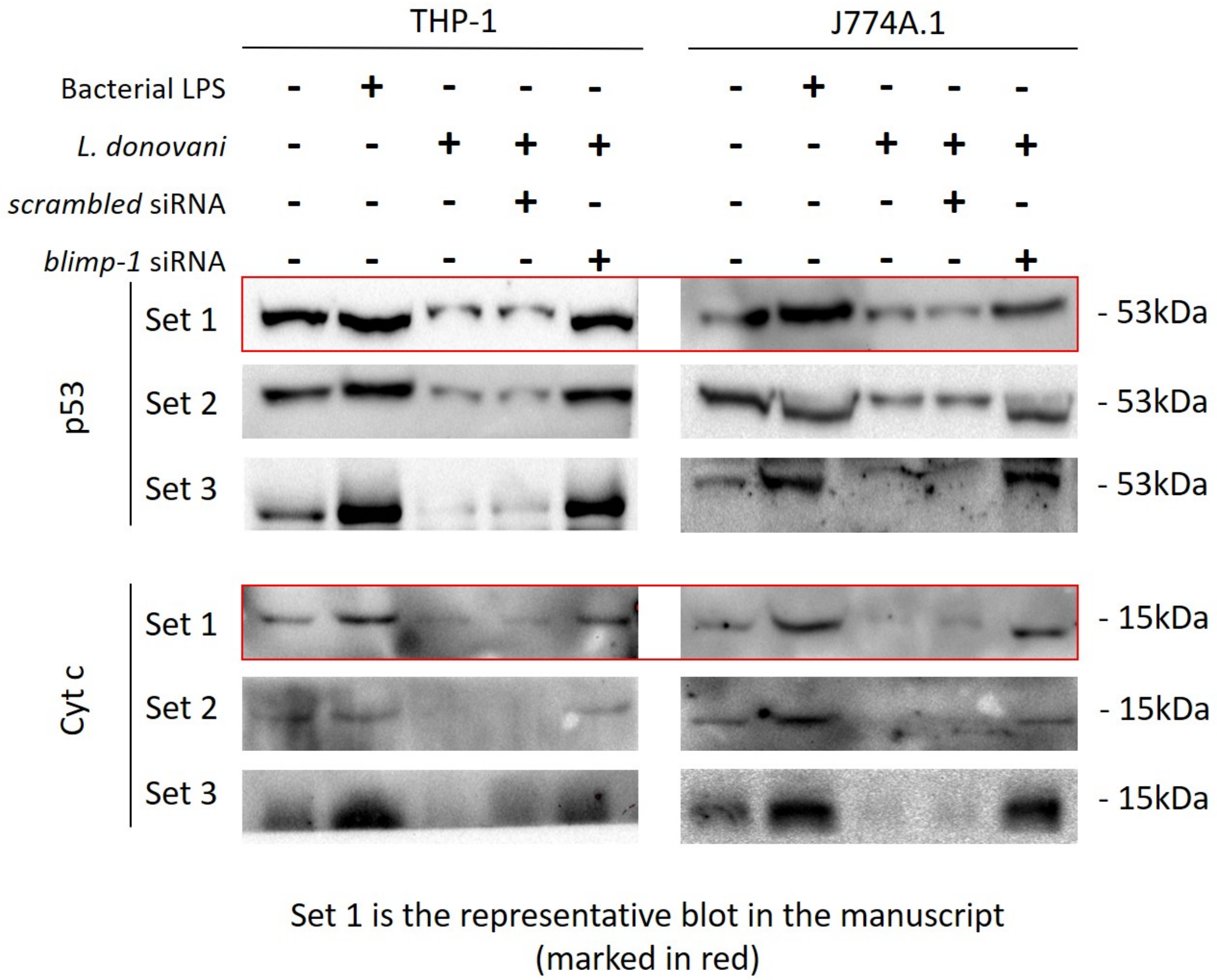

Supp. Fig. 5: Different replicate blots were shown for manuscript figure 2. Red marked (Set 1) was used in the manuscript as representative blot. (Note: n=3)
